# Development and evaluation of CARIES-QC: a caries-specific measure of quality of life for children

**DOI:** 10.1186/s12903-018-0662-8

**Published:** 2018-12-04

**Authors:** Fiona Gilchrist, Helen D. Rodd, Chris Deery, Zoe Marshman

**Affiliations:** School of Clinical Dentistry, University of Sheffield, Claremont Crescent, Sheffield, S10 2TA England

**Keywords:** Child, Dental caries, Quality of life, Qualitative research, Outcome assessment

## Abstract

**Background:**

Existing paediatric oral health-related quality of life (OHRQoL) measures are generic instruments designed to evaluate a range of oral conditions. It has been found that disease-specific measures may be more adept at detecting subtle changes which occur following treatment of the condition in question. Furthermore, existing self-report OHRQoL measures have not involved children at all stages of development of the measure. The aim of this study was to develop a caries-specific measure of quality of life for children.

**Methods:**

The first stage of the study involved a qualitative enquiry with children, aged 5–16 years, to inform the development of the measure. Children generated the potential items, contributed to item reduction and questionnaire design and participated in the testing of face and content validity. The resulting measure was evaluated in a cross-sectional validation study. Ethical approval was granted for the study.

**Results:**

The qualitative study found that children discussed a number of caries-related impacts which affected their daily lives. These were incorporated into a draft measure which was further refined following testing of face and content validity. This resulted in the production of the Caries Impacts and Experiences Questionnaire for Children (CARIES-QC), comprising 16 items and one global question. Two hundred participants with a mean (range) age of 8.1 (5–16) years took part in the further evaluation of CARIES-QC. Four items, which did not fit the Rasch model, were removed from further analysis. The remaining 12 items demonstrated good internal consistency (alpha = 0.9) and the total score showed significant correlations with the number of decayed teeth, presence of pain, pulpal involvement, the Child Perceptions Questionnaire (16-item short form) and the global score (*p* < 0.01, Spearman’s rho).

**Conclusion:**

In conclusion, children’s input allowed the development of a valid and reliable child-centred caries-specific quality of life measure. CARIES-QC can now be used to evaluate which interventions for dental caries are most effective in reducing impacts from the child’s perspective.

**Electronic supplementary material:**

The online version of this article (10.1186/s12903-018-0662-8) contains supplementary material, which is available to authorized users.

## Background

Dental caries remains a global public health concern. A recent systematic review reported that approximately 621 million children have untreated dental caries [1]. Despite its worldwide prevalence, little attempt has been made to seek children’s own accounts of how this disease impacts on their daily lives.

The main focus of studies on the impact of dental caries in children has, not surprisingly, been to determine the prevalence of dental pain in child populations. Whilst pain is clearly an important sequelae of caries, consideration should also be given to the wider psychosocial aspects of this universal disease. Findings from previous studies using oral health-related quality of life measures (OHRQoL) have shown that children with dental caries frequently report functional impacts such as difficulty eating, drinking and pain when brushing teeth [2–6]. In addition to these functional concerns, children with caries also report wider psychosocial impacts related to smiling, playing, difficulty sleeping or relaxing, emotional/social wellbeing and schoolwork [2, 3, 7].

However, the relationship between clinical caries data (e.g. extent) and child OHRQoL has been found to be weak and inconsistent [8–12]. Several explanations have been suggested to account for this finding, including: limitations of existing child measures of OHRQoL [13]; analysis of child populations with relatively low levels of disease, and the effects of socioeconomic status, cultural differences and general health which may all mediate the impacts [11, 14–16].

One of the limitations of existing self-report OHRQoL questionnaires is that they are ‘generic’ measures meaning they are designed to capture the impacts of all oral conditions on children’s lives. Wiebe and co-workers (2003) found that in randomised controlled trials with a true underlying therapeutic effect, disease-specific instruments were more responsive to change in health related quality of life than were generic instruments [17]. Therefore, while generic measures are useful to compare populations and can be used to compare groups with different health conditions, disease-specific measures are more adept at measuring changes in individuals with a specific disease.

Furthermore, inherent limitations of existing self-report measures for evaluating OHRQoL in children with caries, (i.e. Child Perceptions Questionnaire, Child-Oral Impacts on Daily Performances index, and Child Oral Health Impact Profile) have been highlighted by a recent systematic review [13]. Firstly, methods adopted to develop these measures included children only at the latter stages of item development, therefore the included items may not fully reflect the language and range of impacts experienced by children.

Secondly, the measurement properties of the existing measures had not been evaluated according to the required standards suggested by the Consensus-based Standards for the Selection of Health Measurement Instruments initiative (COSMIN) [18]. However, it is acknowledged that the measures included in this 2014 systematic review were all developed prior to the existence of the criteria used to assess them, which may account for some of the deficiencies identified. Since this review was undertaken, further studies have been published which have used item response theory (IRT) methods with existing measures and have recommended changes based on their findings [19–21]. Others have measured responsiveness of the existing self-report measures. However, none of these studies investigated correlations between change scores and the global transitional judgement question to confirm responsiveness, as recommended by the COSMIN group [22–25].

In order to address these acknowledged limitations, a caries-specific quality of life measure, which has involved children at all stages in its development and is sensitive to changes resulting from interventions to manage dental caries is required. Such a measure would have important applications in future clinical trials which seek to prevent or manage caries.

The aim of this study therefore was to develop and validate a caries-specific measure of quality of life ‘CARIES-QC’ for children which could be used to evaluate different approaches for the management of dental caries. The objectives were to:

1. Involve children in the design and content of the measure to ensure that it is meaningful and relevant to them.
2. Evaluate the properties of the measure including validity, reliability and responsiveness.

## Method

**Fig. 1:**
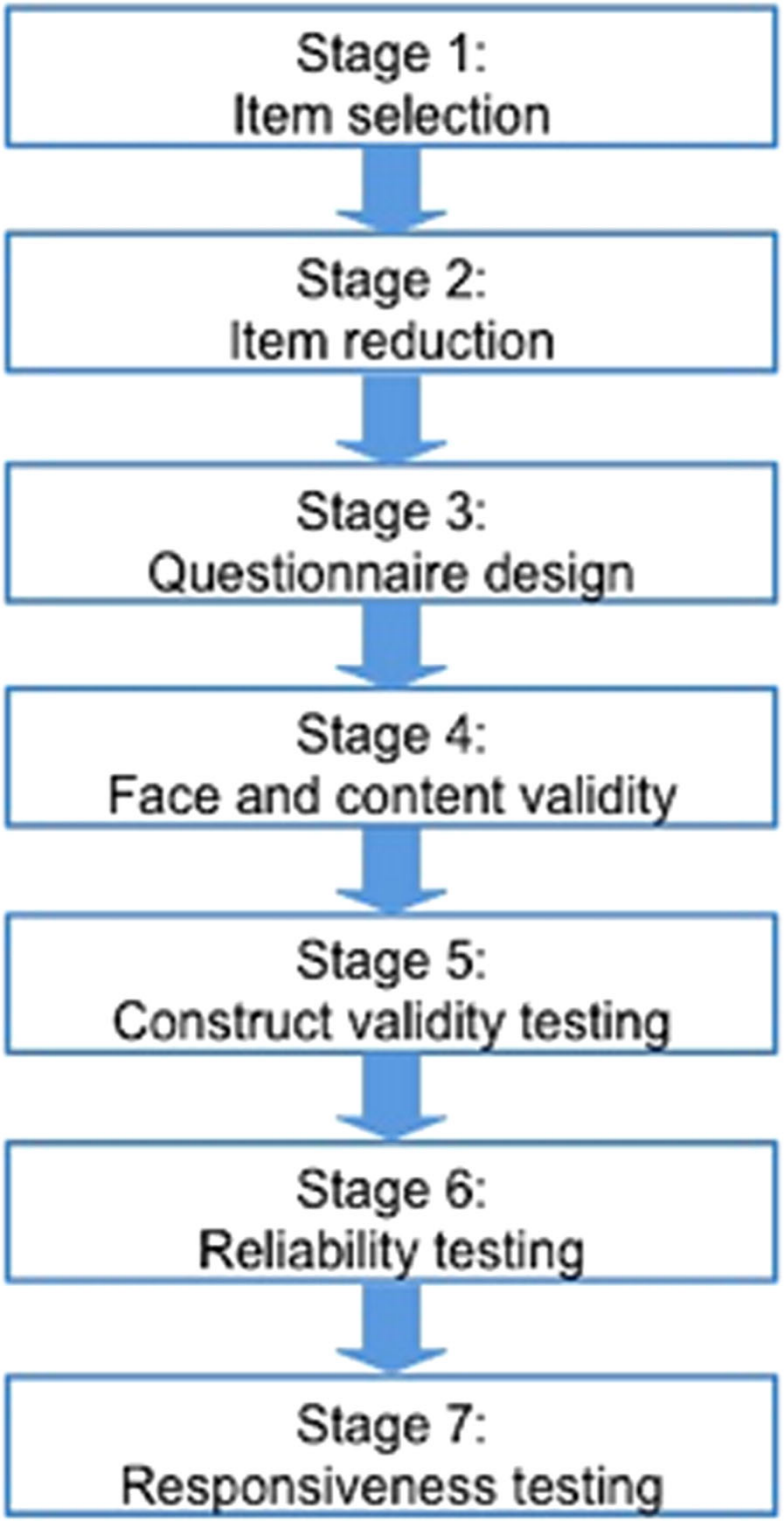
Questionnaire design stages adapted from Guyatt and colleagues (1986)

The measure was developed based on the definition of OHRQoL as proposed by Locker and Allen (2007) to encompass “the impact of oral diseases and disorders on aspects of everyday life that a patient or person values, that are of sufficient magnitude, in terms of frequency, severity or duration to affect their experience and perception of their life overall” [26]. The measure was developed using the robust methodology proposed by Guyatt and coworkers (1986) [27]. A seven-step process was used as recommended, involving: item generation; item reduction; questionnaire design; testing of face and content validity, and testing of validity reliability and responsiveness (Fig. 1). Each of these steps will be described in turn. The methodology was child-centred and the measurement properties were evaluated using both classical test theory and item response theory. Ethical approval was granted by the South Yorkshire Research Ethics Committee (Reference number: 11/H1310/3).

### Development of CARIES-QC

This part of the investigation comprised four stages (Fig. 1). Children aged 5–16 years who could speak English with active caries or who had treatment of dental caries in the preceding year were invited to participate. Clinical and demographic data were collected including: age; gender; ethnicity; postcode (to assign Index of Multiple Deprivation (IMD) score/rank); dmft/DMFT; presence of anterior caries; pain and pulpal involvement. DMFT/dmft was assessed by FG using a combination of clinical and radiographic evidence where available. Caries was judged to be present where caries had extended into dentine either clinically and/or radiographically. The exception to this was that both children with and without caries experience were asked to comment on the questionnaire design and face validity as detailed below. Exclusion criteria included children with pre-existing significant medical conditions, other dental conditions (trauma, dental anomalies), or those with severe learning difficulties who would be unable to participate with the intended activities even with support.

### Stage 1: Item generation

The aim of this stage was to generate potential items for the measure and to identify the language children used to describe impacts that they experienced as a result of dental caries. Children were purposively sampled from both a primary care dental setting (Firth Park Clinic, Sheffield Salaried Dental Services {FPSDS}) and a secondary dental care service (Paediatric Dental Department at the Charles Clifford Dental Hospital {CCDH}, Sheffield) to take part in qualitative focus groups and interviews. Recruitment continued until data saturation was achieved [28]. Further details of this initial qualitative phase in the development of CARIES-QC have been previously reported [29].

The sociology of childhood guided the conduct and analysis of the focus groups and interviews [30]. This approach regards children as capable experts in their own lives and gives them a voice as research participants. In order to achieve this and to prioritise the child’s voice, the analysis took a narrative approach. That is, rather than trying to “verify” what children said, the focus was on how they described their experiences and what they meant to them [31]. A total of 20 children participated in this stage. Interviews (*n* = 15, conducted by FG) and focus groups (*n* = 2 with 5 children, facilitated by FG and ZM) were held with children aged 5–13 years (12 male, 8 female) who had a wide range of clinical presentations. The majority (*n* = 17) were white British and 11 children lived in areas ranked as the most deprived quintile in the UK (Table 2). Details of the children’s clinical presentations are reported elsewhere [29].

Framework analysis was used to classify the data according to themes and categories that emerge from the data. This technique has developed from social policy research to facilitate handling large volumes of data [32]. The analytical approach involved the following stages [33]:1*.* Identifying initial themes; 2. Labelling the data; 3. Sorting the data by theme and 4. Synthesising the data. Further details of this analysis have been published elsewhere [29].

Using this approach, 17 different items, shown in Table 1, were described by children. It was decided that the response options “a bit”, “a little”, “sometimes”, “very much” and “a lot” would be initially adopted as these descriptors had been most commonly used by participants during conversations. The next stage of the project (see below) aimed to verify which of these words would be most suitable in the final response format.

**Table 1:** Items generated from initial interviews and focus groups

Potential items following analysis of interviews	
• Pain (hurts) • Difficulty eating some foods • Having to eat on one side • Getting food stuck in teeth • Being kept awake by pain • Feeling annoyed • Having to take medicine • Pain during toothbrushing • Having to eat more slowly • Having to eat more carefully • Crying • Front teeth looking brown • Feeling tired • Not being able to do schoolwork • Difficulty talking • Feeling grumpy • Not been able to do normal activities	

### Stage 2: Item reduction

The aim of this stage was to ascertain which caries-related items were most relevant to children and to identify if there were any additional items which should be included. During January and February 2014, 25 children attending CCDH who were having treatment for caries, were identified and 22 were recruited to Stage 2 of the project. Previous studies have used samples of over 75 participants for this stage, where there have been a large number of items generated [34–36]. As items were to be deleted from the questionnaire following Rasch analysis, item reduction in this study was more limited and with a smaller sample, allowing in-depth discussion of the potential items with participants. Two children declined to participate as they had become distressed during radiographic examination and one family declined due to time constraints. Characteristics of these participants are shown in Table 2.

**Table 2:** Characteristics of participants in development stage

Study stage	Gender		Mean (range) age (years)	Deprivation quintile				
	Male	Female		1	2	3	4	5
Item generation (*n* = 20)	11	9	9.55 (5.5–13.9)	1	2	4	2	11
Item reduction (*n* = 22)	9	13	9.40 (4.8–15.7)	3	1	4	2	12
Questionnaire design (*n* = 10)	7	3	9.9 (6.4–15.5)	4	3	3	0	0
Face validity only (*n* = 4)	1	3	10.84 (8.8–11.96)	2	2	0	0	0
Face and content validity (*n* = 4)	4	0	9.45 (6.45–12.5)	0	1	0	0	3
Content validity only (*n* = 21)	8	13	8.24 (5.19–14.35)	1	2	2	4	13
Overall (*n* = 81)	29	32	9.17 (4.82–15.72)	11	11	13	8	46

Deprivation quintile: 1= least deprived and 5= most deprived

Children were shown cards depicting the items identified from the item generation stage (Table 1), and they were asked to indicate which ones they had experienced, if any. They were also invited to say which item/s had been the most severe and which they had experienced the most frequently. The mean number of caries-related impacts was six, with a range of zero to 12. Only one child had not experienced any of the impacts listed, and indeed had not suffered any symptoms or negative effects as a result of his caries. ‘Pain’, ‘getting food stuck in their teeth’ and ‘having to eat carefully’ were the items which had affected most children (*n* = 16, 72.7%). ‘Pain’ was also reported as being the most severe impact. In contrast, ‘getting food stuck’ was the impact which reportedly occurred most frequently. Seven children (31.8%) could not identify what had been the worst impact and seven children (31.8%) could not identify which impact had happened most frequently.

‘Having difficulty talking’ and ‘not been able to do normal activities’ were endorsed the least. Since these two items were not chosen as having the highest impact or occurring with the greatest frequency, it was decided to omit them from the measure as per Guyatt and co-workers’ recommendations [27]. The remaining 15 items were taken forward to the next stage for evaluation of face and content validity. Following the completion of this stage, draft questionnaires were developed to prompt discussions in the questionnaire design stage.

### Stage 3: Questionnaire design

This stage aimed to discover which words were most suitable as the response format, to ascertain children’s preferences for the question layout and design and to check the wording of the instructions. Ten participants were recruited in January 2014 either from CCDH or a local general dental practice (Table 2). These one-to-one interactive sessions were audiotaped (Olympus Digital Voice Recorder WS-812) and transcribed verbatim. Recruitment continued until data saturation was achieved. Children were asked which of the descriptors “not at all”, “a bit”, “a little” “a lot”, “sometimes” and “very much” they preferred and they were asked to rank them from worst to best. Most children felt that “a bit” and “a lot” were words that they used most often with their friends and all were able to consistently rank these in the correct order.

Children were then asked to complete six pilot questions using two different formats, to identify their preferences (Additional file 1). Several suggestions were made to improve the clarity and wording of the questions and instructions. These suggestions can be viewed in Additional file 1.

In keeping with the children’s suggestions, the final measure therefore invited participants to circle one of three response options: “not at all”, “a bit” and “a lot”.

### Stage 4: Face and content validity

This stage aimed to ensure that no important items had been omitted and that children interpreted the questions as intended. A total of eight children (with and without caries) took part in the analysis of face validity and 25 children with active caries or caries experience took part in analysis of content validity, including two children with learning disabilities. Recruitment continued until saturation was achieved. Participant characteristics are shown in Table 2. Children attending CCDH or FPSDS in March 2014 were consecutively recruited to this stage of the investigation. All children who were invited to participate agreed to take part. The testing took an iterative approach, with amendments made during the period of testing to allow these changes to be subsequently evaluated by other children.

Children were asked to complete the questionnaire and discuss why they had chosen their answer, so that their comprehension of the question could be confirmed. This approach has previously been used with both adults and children to explore face and content validity of health-related quality of life measures [37, 38]. Several insightful suggestions were made by participants. These included the addition of a question relating to being able to see holes in teeth; adding “black” as well as “brown” to reflect tooth colour and “grumpy” was changed to “cross” as this was more indicative of how they felt. Further minor amendments were made but no further items were added and no items were felt to be irrelevant.

The global question was based on the one used in the Child Perceptions Questionnaire: “Overall, how healthy are your teeth?” [36] with the response options “Not healthy”, “A bit healthy” and “Very healthy”. This was modified following testing as it became clear that the participants were not answering this question as expected. As this question had poor face validity, two further global questions were tested with children and the preferred one included in the final version of the measure.

The resultant 16-item measure took approximately 2–5 min to complete depending on the reading ability of the child, with some younger children requiring assistance to read it. CARIES-QC, including the instructions which are intended to be read with a parent, has a Simple Measure of Gobbledygook (SMOG) index of 5.3 and a Flesch-Kincaid grade score of 1.5 indicating a reading age of 6–10 years (http://www.editcentral.com/gwt1/EditCentral.html).

### Evaluation of the measure

As recommended by the COSMIN group, Rasch analysis, a form of item response theory, was used in the development of CARIES-QC. Rasch provides one mathematical model to guide the production of a linear scale which enables accurate calculation of change scores and therefore is suitable for measurements relating to health interventions [39]. The measure was also evaluated using classical test theory. This part of the investigation comprised three stages (Fig. 1).

### Stages 5, 6 and 7: Validity, reliability and responsiveness

#### Recruitment

A consecutive sample of participants was recruited from new patient clinics in the paediatric dental department of CCDH and from FPSDS. Participants who understood spoken English aged 5–16 years with active dental caries were recruited between July 2014 and January 2015. The same exclusion criteria were applied as used during the development stage and the same clinical and demographic data were collected. Parents were advised that they could help the child to read the questions if required but that it was the child’s responses which were desired.

#### Questionnaires

Data were collected at three time points where possible. These were:

- Baseline (T0), for example, at a new patient clinic appointment
- Prior to the start of dental treatment to allow test-retest reliability (T1)
- Following a course of dental treatment for the management of dental caries (T2)

### Baseline responses (T0)

At T0, participants were asked to complete two questionnaires: CPQ_11–14_-ISF:16 and CARIES-QC. The CPQ_11–14_-ISF:16 contains 16 items and two global questions [40]. Participants are asked how often (never, once or twice, sometimes, often, every day or almost every day) they have experienced the listed impacts during the past 3 months. The questions are scored on a 5-point Likert scale from 0 to 4, with increasing score indicating increasing frequency of impact (total score possible ranges from 0 to 64). The higher the score, the poorer the rating of OHRQoL. This version of the short form of the CPQ_11–14_ has been used successfully in children aged 5–8 years despite being originally developed for older children [41].

The initial version CARIES-QC contains 16 items and one global question. The items are scored on a 3-point Likert scale (“Not at all”; “A bit” and “A lot”) and scored 0–2 with increasing score indicating increasing severity of the impact. A higher total score indicates increased impact. Participants were randomised (randomizer.org) to receive either Questionnaire A (CARIES-QC followed by CPQ_11–14_-ISF:16) or Questionnaire B (CPQ_11–14_-ISF:16 followed by CARIES-QC) to control for order effect. This approach was adopted to ensure that the order in which the questionnaires were administered had no effect on the overall scores for each measure. For test-retest reliability, participants who returned for a prevention visit prior to treatment were asked to complete CARIES-QC once more. This questionnaire, used for test-retest reliability and post-treatment, contained a supplementary global transitional judgment (GTJ) question which asked whether the participant’s teeth felt “the same”, “better” or “worse” compared to the time of the previous administration. Only those who reported that their oral condition had remained stable were included in analysis of test-retest reliability.

### Data analysis

The RUMM2030 (RUMM Laboratory Pty Ltd., Perth, Australia) software was used for all Rasch analyses, which were based on the unrestricted or partial credit model [42, 43]. All other analyses were undertaken using SPSS 20 (IBM, New York, United States). The Index of Multiple Deprivation score/rank (2010) was calculated using GeoConvert applied to the participant’s postcodes [44].

### Missing items

Where more than two questions were unanswered in CARIES-QC, the participant was eliminated from further analysis. Where more than two values were missing from CPQ_11–14_-ISF:16, the individual questionnaire was excluded from further analysis. Where less than two missing values were noted, the missing value was replaced with the computed mean of the participant’s completed items [45]. This was not required for the Rasch analysis, as the calculations take account of missing values and therefore mean values were not substituted.

### Analysis using the Rasch model

Rasch analysis was performed for the data pertaining to the initial administration of CARIES-QC. According to this method, the items chosen for the final measure should be unidimensional, free from differential item functioning (DIF), i.e. they function in the same way across groups, and fit the model expectations [46]. A sample size of at least 150 participants is recommended to give 99% confidence that the estimate is within 0.5 logits [47].

The measure was tested with the unrestricted or partial credit model, using the method suggested by Tennant et al. (2007) involving [46]:

1. Category discrimination, this analysis seeks to assess whether participants are able to discriminate between the different response options. Where these were found to be disordered, the question was removed, as CARIES-QC has a 3-point response scale and collapsing of adjacent categories is not appropriate.
2. Local dependency, this was deemed to be present if residual correlations were greater than 0·2 above the average residual correlation [48].
3. DIF was analysed by age (5-7 years, 8–11 years and 12–16 years), gender, ethnicity (White British or non-White British) and deprivation (three equal groups created from IMD scores).
4. Item fit to the model, if the data fit the Rasch model, each item and person fit residual should be within the range ± 2·5 and the mean item and person fit statistics should be close to zero with a standard deviation of one [48]. Finally, the individual items and summary chi-square interaction statistics should be non-significant (*P* > 0·05), although these are subject to Bonferroni adjustment based on the number of items. Strict unidimensionality was then examined using an independent t-test on two subsets of items identified using principal component analysis of the item residuals.
5. Reliability was evaluated using the Person Separation Index (PSI). This is equivalent to Cronbach’s alpha, however the logit value is used instead of the raw score. It is interpreted in the same manner, i.e. a value of greater than 0·7 indicates good internal consistency.

As CARIES-QC focuses on attributes which are not directly measurable, such as pain and emotional impacts, the raw score will only be indicative of a rank along the scale, this precludes simple addition and subtraction of raw scores [49, 50]. In order, to use the raw score to accurately measure change, conversion to an interval level scale is required. This can be achieved by transforming the ordinal score to a logit score [50]. All further analyses, where appropriate, were based on the scale created from this analysis.

### Interpretability

Floor and ceiling effects were defined as present if more than 15% of participants reported the best or worst possible score [51]. The mean, range and standard deviation (SD) of scores were calculated for all subgroups (gender, age, ethnicity and deprivation group). Independent t-tests and one-way ANOVA were used to test for differences between the transformed interval CARIES-QC score (CIS) according to clinical and demographic variables as these data were normally distributed.

### Internal consistency, reliability and construct validity

In addition to the Rasch analysis, to allow comparison with similar scales, Cronbach’s alpha was calculated for both CARIES-QC and CPQ_11–14_-ISF:16. Cronbach’s alpha of > 0.7 is accepted as indicating a homogenous scale [51].

The intraclass correlation coefficient (ICC) was calculated for all participants who remained stable between T0 and T1. A value > 0.7 is deemed to indicate acceptable internal reliability [51].

A one-way ANOVA was performed to check for order effect (between Questionnaire A and B). Construct validity was tested using the appropriate bivariate correlations between CARIES-QC total score and: CPQ_11–14_-ISF:16 total score; the presence of pain; pulpal involvement; anterior caries; total number of carious teeth; total caries experience and the global scores of both CARIES-QC and CPQ_11–14_-ISF:16. To assess convergent construct validity it was hypothesised that there would be positive correlations with CPQ_11–14_-ISF:16 (especially for oral symptoms, functional and emotional wellbeing domains) and the global questions from both CARIES-QC and CPQ_11–14_-ISF:16. It was also hypothesised that there would also be positive correlations between CARIES-QC total score and clinical data (the total number of carious teeth, the presence of pain and pulpal involvement), thereby assessing its concurrent construct validity. These hypotheses were based on the information obtained from the qualitative data described elsewhere [29].

### Responsiveness

This was analysed using correlations between the mean change score at T2 and the global change score reported by the participant (− 1 = worse, 0 = same, + 1 = better) as recommended by the COSMIN group [18]. It was hypothesised that change score (difference between T0 and T2) would correlate with the global score, with those feeling there had been an improvement in their condition having a lower total score than those who felt they had stayed the same or felt worse. It was also hypothesised that those who felt that they had improved would have lower mean scores than those who felt their dental condition had deteriorated or remained unchanged following treatment.

## Results

### Stages 5, 6 and 7: Validity, reliability and responsiveness

Of those who were approached to participate in this stage, only one child declined, giving a response rate of 99%. A total of 202 participants were recruited over the 7-month period. Two (1%) participants did not complete CARIES-QC and therefore were eliminated from further analysis. There were 95 (47.5%) males and 105 (52.5%) females with a mean age of 8.1 (range = 5.0–16.0) years. The majority of children identified themselves as being white British (65%, *n* = 130). The majority (59.5%, *n* = 119) of participants lived in the most deprived quintile according to UK national IMD ranking scores [52]. Further demographic information is shown in Table 3.

**Table 3:** Demographic characteristics of the participants (*n* = 200)

Variable	Proportion	Number
Gender		
Male	47.5%	95
Female	52.5%	105
Ethnicity		
Asian background	15.5%	31
Black background	2.5%	5
Mixed background	4.5%	9
White British background	65.0%	130
Other background	4.5%	9
Unknown background	8.0%	16
Socioeconomic status		
Most deprived	59.5%	119
More deprived	18.5%	37
Average	10.0%	20
Less deprived	6.5%	13
Least deprived	5.5%	11

Twenty-two (11%) children were in the permanent dentition, 72 (36%) in the primary dentition and 106 (53%) were in the mixed dentition. Further details of caries experience are shown in Table 4. Anterior caries was present in 41 (20.5%) of the participants. Pulpal involvement was present in 160 (80%) and pain reported in 145 (72.8%) of participants.

**Table 4:** Caries experience of included participants (*n* = 200)

	Minimum	Maximum	Mean (SD)
dmft	0	16	6.24 (3.45)
Number of carious primary teeth	0	14	5.74 (3.40)
Number of missing primary teeth	0	10	0.27 (1.15)
Number of filled primary teeth	0	4	0.22 (0.69)
DMFT	0	13	1.57 (2.18)
Number of carious permanent teeth	0	9	1.38 (1.77)
Number of missing permanent teeth	0	4	0.05 (0.38)
Number of filled permanent teeth	0	7	0.13 (0.74)
Total number of carious teeth	1	14	6.01 (3.27)
Total number of missing teeth	0	10	0.27 (1.12)
Total number of filled teeth	0	7	0.28 (0.89)

*SD* Standard deviation

*dmft* Total number of decayed, missing and filled primary teeth

*DMFT* Total number of decayed, missing and filled permanent teeth

### Missing data

At baseline, a total of 11 (5.5%) participants had missing values for CARIES-QC and 17 (8.5%) for CPQ_11–14_:ISF:16. Three (1.5%) and nine (4.5%) participants had greater than two missing values in CARIES-QC and CPQ_11–14_:ISF16 respectively and were therefore eliminated from the following analyses with the exception of the Rasch analysis. Those with more than two missing responses had generally omitted entire pages. Missing values were replaced with the participant’s overall mean value. Where participants had omitted the global question, this was not replaced by a mean value and therefore correlations with the global score were not undertaken for these participants. There were no missing values at T1 and only one missing value at T2 in CARIES-QC. The missing value at T2 was replaced with the participant’s overall mean value.

### Analysis using the Rasch model

**Fig. 2:**
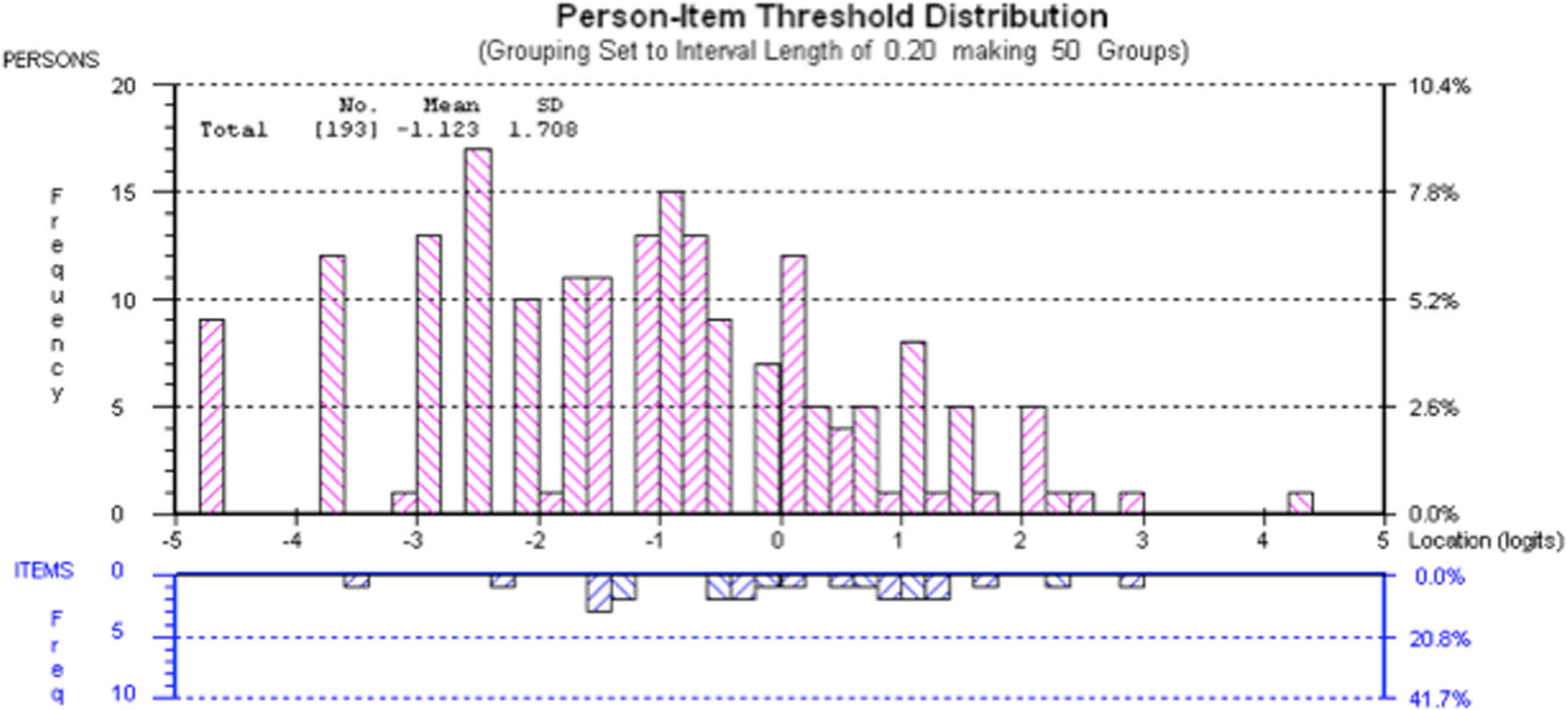
Targeting of CARIES-QC. The upper section of the graph shows the distribution of participants and the lower part the distributions of thresholds (category transitions) of the items. The x-axes display the location (severity of impact) of the participants and the item location (difficulty) of the item thresholds. The y-axes show the frequency of item thresholds and participants

Two hundred participants were included in the Rasch analysis. One item (“feeling tired”) had a disordered threshold and three items demonstrated misfit to the model (“taking medicine”, “front teeth looking brown or black” and “being able to see holes”). No DIF was observed. Seven participants did not fit the Rasch model and were therefore removed from further Rasch analysis. This resulted in acceptable fit statistics (Additional file 2). The mean person location is − 1.12 when the items are centred on zero. This demonstrates that the scale is targeted to a population with slightly more impacts than the participants in the present study, which may be due to the number of participants who reported no or low levels of impacts. Figure 2 shows the person-item threshold map and demonstrates that participants are distributed in a similar pattern to the items, indicating that the items measure the impacts of caries along the construct from least to most. As the items fit the Rasch model, a transformation from the raw score (possible range 0–24) to interval scaling is shown in Table 5.

**Table 5:** Transformation of raw (ordinal) score to interval score

Raw score	Interval score
0	0
1	2.63
2	4.50
3	5.84
4	6.90
5	7.80
6	8.60
7	9.32
8	10.00
9	10.64
10	11.26
11	11.86
12	12.45
13	13.03
14	13.62
15	14.22
16	14.84
17	15.48
18	16.17
19	16.92
20	17.76
21	18.75
22	19.96
23	21.65
24	24.00

### Interpretability

As four items had been removed following previous analysis. The following analyses are based on the remaining 12-items. One hundred and ninety-seven participants were included in the analysis of CARIES-QC, three were excluded as they had omitted more than two questions.

At baseline, nine (4.6%) participants scored the lowest possible score (0) and one (0.5%) the highest (24), which is within an acceptable range for floor and ceiling effects. The mean (SD; range) raw score was 8.08 (5.52, 0–24) and the CIS mean score was 9.34. Mean CIS for the different subgroups are shown in Table 6. Statistically significant higher mean scores were found for children who reported pain (CI = 2.63–5.19; *p* = < 0.001) compared those who had not. This was also true for those who had anterior caries (CI = 0.16–3.20; *p* = 0.03) when compared with those without anterior caries and those who had pulpal involvement (CI = 0.79–3.82; *p* = 0.03) when compared with those without pulpal involvement. A statistically significant difference was found between ethnic groups (CI = − 2.74 to − 0.13; p = 0.03), with those from non-white British backgrounds having higher scores than those from white British backgrounds. This was despite there being no significant difference between the number of carious teeth, the presence of pain or pulpal involvement between subgroups.

**Table 6:** Mean, range and standard deviation of CARIES-QC baseline scores

Participants/subgroups		CARIES-QC interval score		
Participants	Number (%)	Mean score	Range	SD
Overall	197	9.34	0–24	4.44
Gender				
Female	103	9.71	0–24	4.59
	(52.3%)			
Male	94	9.05	0–18.75	4.28
	(47.7%)			
Age group				
5–7 years	114	9.82	0–24	4.51
	(57.9%)			
8–11 years	66	8.91	0–18.75	4.55
	(33.5%)			
12–16 years	17	8.90	2.63–16.17	3.49
	(8.6%)			
Pain				
Yes	142	10.49a**	0–24	4.09
	(72.1%)			
No	55	6.57	0–17.76	4.10
	(27.9%)			
Pulpal involvement				
Yes	157	9.86b*	0–24	4.26
	(79.7%)			
No	40	7.56	0–17.76	4.72
	(20.3%)			
Anterior caries				
Yes	41	10.72c*	0–24	4.71
	(20.8%)			
No	156	9.04	0–19.96	4.32
	(79.2%)			
Ethnicity				
White British background	130	8.91	0–18.75	4.29
	(66.0%)			
Other background	53	10.57d*	0–24	4.82
	(34.0%)			
Deprivation				
Deprivation group 1 (lowest)	67	9.20	0–24	4.92
	(34.0%)			
Deprivation group 2 (middle)	65	9.93	0–19.96	4.35
	(33.0%)			
Deprivation group 3 (highest)	65	9.04	0–17.76	4.02
	(33.0%)			

*SD* Standard deviation, a**=children who reported pain had significantly higher mean CARIES-QC interval score than those who did not report pain (CI = 2.63–5.19; *p* < 0.01); b*=children who had pulpal involvement had significantly higher mean CARIES-QC interval score than those who had no pulpal involvement (CI = 0.79–3.82; *p* < 0.05); c*=children with anterior caries had significantly higher mean CARIES-QC interval score than those who did not have anterior caries (CI = 0.16–3.20; *p* < 0.05); d*=children from non-white British backgrounds had significantly higher mean CARIES-QC interval score than those from white British backgrounds (CI = −2.74 to − 0.13; *p* < 0.05)

At T1 (*n* = 71), the mean (SD; range) raw score was 6.30 (4.87; 0–20) with three (4.5%) participants scoring the lowest possible score. The CIS mean (SD; range) score was 7.97 (4.09; 0–17.76). For the 31 participants who reported that they had experienced no change since T0, the mean (SD; range) CIS was 7.96 (3.55; 2.63–16.17). This contrasted with a lower mean (SD; range) CIS of 7.08 (4.39; 0–17.76) in those who reported that their teeth felt better (*n* = 33) and a higher mean (SD; range) CIS of 12.14 (2.22; 8.6–14.84) in those who felt their teeth had deteriorated (*n* = 7).

The impact which was most commonly reported by participants at baseline was “food stuck” (*n* = 173, 87.5%) and the least reported impact was “interfering with schoolwork” (*n* = 34, 17.3%). The majority of items were reported by greater than 50% of participants. Interestingly, there were 62 (31.5%) children who answered that they had experienced no pain. However, 53 (85%) of these children reported other impacts related to their caries experience with CARIES-QC total scores ranging from 1 to 11 (mea*n* = 2.97). This compared with scores of 1–24 (mea*n* = 10.24) for those answering that they either had “a bit” or “a lot” of pain. A statistically significant difference in mean CIS was found between the pain and non-pain subgroups (*p* = 0.00; CI = − 4.89 to − 0.84). At baseline, 64.5% (*n* = 127/196) reported that their teeth were “a bit” or “a lot” of a problem with respect to the global question.

### Internal consistency, reliability and construct validity of CARIES-QC

Cronbach’s alpha was found to be 0.9. This did not increase if any items were deleted, as would be expected following the Rasch analysis which was used to confirm internal consistency of the items. Item total correlations ranged from 0.509 (food stuck) to 0.719 (eating carefully).

A total of 70 participants participated in the test-retest analysis at a mean (range) of 29 (3–127) days from T0. Thirty-one participants reported that their condition had remained stable. Those participants who had reportedly remained stable had similar clinical and demographic characteristics at baseline to the participants at T0. The ICC was 0.7.

No order effect was present (*p* = 0.732). A strong correlation was found between CARIES-QC total and the global question (*r* = 0.734) (*p* < 0.01). Weaker but significant (*p* < 0.01) correlations were found between the total CARIES-QC score and pain (*r* = 0.392), the total number of carious teeth (*r* = 0.188) and with the presence of pulpal involvement (*r* = 0.187). Further details are shown in Additional file 3. Significant (p < 0.01) correlations were also found between CARIES-QC total score and the total CPQ_11–14_-ISF:16 score (*r* = 0.733), the global oral health question from CPQ_11–14_-ISF:16 (*r* = 0.291) and life overall rating from CPQ_11–14_-ISF:16 (r = 0.392). Statistically significant correlations were found with CARIES-QC total score and all domains of CPQ_11–14_-ISF:16 with the highest correlation being between the oral symptoms (*r* = 0.646), functional limitations (*r* = 0.665) and emotional wellbeing (*r* = 0.630) domains.

### Responsiveness

Data from 43 participants were available following a comprehensive course of treatment (T2). These participants had a similar profile to those included at TO, although a higher proportion of these post-treatment children had pulpal involvement at baseline. Their mean (range) age was 7.86 (5.33–12.56) and 40.5% were male. The mean (range) dmft was 6.18 (1–12) and mean (range) DMFT was 1.04 (0–4).

Of the children who participated at T2, 34 (79.0%) reported an improvement since baseline, 6 (14.0%) reported no change and 3 (7.0%) reported a deterioration in their oral condition. The mean (SD; range) raw score was 4.09 (3.96; 0–16), with six participants (14.3%) scoring the lowest possible score. Extraction of teeth under general anaesthesia (GA) was the most common treatment provided (*n* = 22, 51.2%). The second most frequent treatment modality was provision of preformed metal crowns using the non invasive Hall Technique [53] (provided prior to the GA) in combination with XGA (*n* = 10, 23.3%). A total of 32 (74.4%) participants had some of their treatment provided under GA. The mean (range) time between the final treatment session and T2 and between T0 and T2 was 66.8 (0–241) days and 138.23 (48–287) days respectively.

Impacts were reported in relation to all items following treatment. The most frequently reported impact at T2 was “food stuck” (*n* = 27, 62.8%), followed by “hurts” (*n* = 17, 39.5%). Responses of “a bit” or “a lot” to the CARIES-QC global question accounted for 30.2% (*n* = 13) answers. With the exception of the item “interfering with schoolwork”, the number of children who reported experience of each impact decreased between baseline and follow-up in those who reported an improvement. For those who reported improvement, the item “annoyed” was seen to have undergone the greatest reduction in frequency of reporting between baseline and follow-up (60.6%), followed by “hurts” (45.5%) and “eating on one side” (42.5%). Comparisons with baseline data are shown in Table 7.

**Table 7:** Number and proportion of participants responding positively (“a bit” or “a lot”) to each item in CARIES-QC following treatment at baseline and follow-up

Item and response	Proportion (number) with impact at baseline (*n* = 197)	Proportion (number) with impact at follow-up (*n* = 43)
Food stuck	*87.8% (173)*	*62.8% (27)*
A bit	55.3% (109)	51.2% (22)
A lot	32.5% (64)	11.6% (5)
Hurts	*68.5% (135)*	*39.5% (17)*
A bit	51.3% (101)	37.2% (16)
A lot	17.3% (34)	*2.3% (1)*
Eating on one side	*62.4% (123)*	*32.6% (14)*
A bit	36.5% (72)	16.3% (7)
A lot	25.9% (51)	16.3% (7)
Cried	*61.4% (121)*	*32.6% (14)*
A bit	47.7% (94)	27.9% (12)
A lot	13.7% (27)	4.7% (2)
Annoyed	*59.9% (118)*	*18.6% (8)*
A bit	40.6% (80)	16.3% (7)
A lot	19.3% (38)	*2.3% (1)*
Eating carefully	*56.3% (111)*	*37.2% (16)*
A bit	38.1% (75)	30.2% (13)
A lot	18.3% (36)	7.0% (3)
Difficult to eat some foods	*56.3% (111)*	*37.2% (16)*
A bit	46.7% (92)	34.9% (15)
A lot	9.6% (19)	2.3% (1)
Eating slowly	*44.2% (87)*	*27.9% (12)*
A bit	33.5% (66)	18.6% (8)
A lot	10.7% (21)	9.3% (4)
Brushing teeth	*42.6% (84)*	*18.6% (8)*
A bit	32.0% (63)	18.6% (8)
A lot	10.7% (21)	0
Feeling cross	*40.6% (80)*	*13.9% (6)*
A bit	28.4% (56)	11.6% (5)
A lot	12.2% (24)	2.3% (1)
Kept awake	*32.5% (64)*	*11.6% (5)*
A bit	27.4% (54)	9.3% (4)
A lot	5.1% (10)	2.3% (1)
Interfering with schoolwork	*17.3% (34)*	*11.6% (5)*
A bit	14.7% (29)	7.0% (3)
A lot	2.5% (5)	4.7% (2)
Global question	*64.5% (127)*	*30.2% (13)*
A bit	49.0% (96)	27.9% (12)
A lot	15.7% (31)	2.3% (1)

Italicised figures indicate the overall proportion (number) responding positively to each item

The mean (SD; range) raw score for those who reported an improvement (*n* = 34) was 2.94 (2.89; 0–14) compared to 6.67 (3.67; 2–12) for those who reported no change (*n* = 6) and 12.33 (3.51; 9–16) for those who felt they were worse following treatment (*n* = 3). The mean difference in CIS between baseline and follow-up for those who felt they had improved was minus 4.42 (range = minus 12.45–plus 2.76; SD = 3.62), thus indicating a MID of 4.42 points. There was a statistically significant difference between the mean score at baseline and follow-up in those who reported an improvement (CI = 3.16–5.65; *p* = 0.00). A strong statistically significant correlation (CI = 3.16–5.66; *p* = 0.01) was found between the GTJ and the CIS change score (*r* = 0.438, Pearson correlation). Comparisons between the groups can be seen in Table 8.

**Table 8:** Mean (range) and change scores calculated using CARIES-QC interval scores (*n* = 42)

Reported condition at follow-up	Mean (range) CARIES-QC interval score at baseline	Mean (range) CARIES-QC interval score at follow-up	Mean (range) change score
All follow-up participants (*n* = 43)	9.46 (2.63–19.96)	5.99 (0–5.48)	−3.48 (minus 12.45–4.10)
Improved (*n* = 34)	9.33 (2.63–19.96)	4.89 (0–13.62)	−4.42 (minus 12.45–2.76)
Unchanged (*n* = 6)	8.32 (4.5–12.45)	8.74 (4.5–12.45)	0.42 (minus 1.52–4.10)
Deteriorated (*n* = 3)	13.44 (11.86–14.84)	12.86 (10.64–15.48)	−0.58 (minus 1.22–0.64)

## Discussion

This study aimed to develop and validate a caries-specific measure of OHRQoL for children. This was achieved through a systematic approach as recommended by Guyatt and colleagues involving an item response theory stage [54].

### Development of the measure

The first stage sought to explore children’s experiences of having dental caries. Children were found to be capable and willing to discuss the impacts they had experienced. Children generally discussed the impacts they had experienced in terms of their severity, rather than the frequency with which they occurred. This is an important finding as some of the existing measures of OHRQoL (CPQ and COHIP) rely on a frequency-based response format which does not appear to reflect how children describe their impacts. This tendency to discuss severity rather than frequency has been found by others who have utilised children’s preferences to develop response formats [55, 56].

Conducting the analysis of face and content validity gave an insight into how children read and answer questions. The analysis of face validity was essential as it revealed problems with the way some questions were worded. In particular, the way children answered the original global question “Overall how healthy are your teeth?”, was interesting as it appeared to have little to do with the condition of their teeth or the symptoms they had experienced. It should be noted that this wording is similar to that used in the global question relating to oral health in CPQ. This may explain why previous studies have found relatively low correlations between the global oral health question in CPQ and total CPQ score in children with caries [9, 10, 12, 36, 57–59]. In the present study, however, there was a strong (*r* = 0.734) correlation between the global oral health question in CARIES-QC and the total CARIES-QC score. This was not seen with the CPQ global oral health question and may indicate that although the global questions of CARIES-QC and CPQ both seek to address the same construct, they do not appear to ask the same question.

### Evaluation of the measure

Children as young as 5 years of age were able to complete CARIES-QC, with their parents or one of the research team helping them to read the questions. This concurs with previous observations that five-year-old children are able to reliably report their health-related quality of life given the opportunity to do so using an age-appropriate instrument [60].

Construct validity was tested using correlations with the total CARIES-QC score and clinical data, the global questions of CARIES-QC and CPQ_11–14_-ISF:16 total score. CARIES-QC had stronger correlations with clinical data than CPQ_11–14_-ISF:16 indicating that it may be more sensitive to assessing those impacts specifically associated with dental caries. CPQ_11–14_-ISF:16 was designed to be generic and be able to evaluate the impacts of a variety of oral conditions, thus it may be that some of the included items are irrelevant to children with caries.

Rasch analysis allowed a unidimensional measure to be produced and identified items which did not fit the mathematical model. Indeed, the question relating to taking medicine, was one for which many children seemed to seek confirmation from their parents. Children appeared to be unsure if they had taken medicine for problems with their teeth and it may be that children are often given analgesia for other problems and therefore cannot remember why they were given it. This question was subsequently removed following Rasch analysis, as it had poor fit statistics, indicating that, it did not work as intended. A similar finding occurred for the items relating to aesthetics. This in hindsight is perhaps not surprising as these aesthetic aspects were identified as a separate theme in the qualitative analysis [29].

A three-point Likert scale was adopted as the response format for CARIES-QC. This approach is in keeping with other studies which have used a three-point scale [61, 62]. However, it is acknowledged that whilst younger children may express a preference for a three-point scale, older children may prefer more options [63]. However, as CARIES-QC was designed to be used over a wide age range and with children who may have low literacy levels or where English may not be their first language, it was decided that a three-point scale would reduce participant burden and allow participation from all demographic groups. With the exception of one question (“feeling tired”), Rasch analysis demonstrated that children were able to distinguish between the three different response options. There is some debate about the number of response options which should be used, with some suggesting sensitivity increases with increasing number of options, whilst others argue that reliability is increased with fewer response options [64]. It has been found that the number of response options may have less of an effect where the items are homogenous, such as in a unidimensional scale [64]. Despite the use of a three-point scale, CARIES-QC was able to differentiate between participants with differing clinical presentations and to evaluate changes following treatment.

For those children reporting an improvement following a course of treatment, the impact which decreased most was “feeling annoyed” in those who reported an improvement. Reduction in this emotional aspect warrants further thought, as one might have expected functional impacts to be most improved following treatment. It may reflect how children perceive chronic dental pain: some children with extensive dental caries do not complain of pain, perhaps because they have had chronic pain for many years and do not recognise this as pain when compared to more acute symptoms. Therefore, a more accurate description from their perspective may be that their teeth are less “annoying”.

This finding also demonstrates the value of including all aspects that are important to patients and not just focusing on functional aspects. Large reductions were also found in the impacts “crying”, “hurts”, “brushing teeth”, “eating on one side” and “feeling cross”, demonstrating the breadth of impacts which were perceived to have reduced following dental treatment. Not surprisingly, given that the majority of patients had multiple teeth extracted as part of their treatment, improvements relating to eating were less marked.

As CARIES-QC is a disease-specific QoL measure, it contains items which are most relevant to children with caries. Thus it is more sensitive to any specific changes which may occur following intervention, as the “noise” from irrelevant questions is reduced [17]. Despite the limited number of participants included in responsiveness evaluation stage, changes which correlated with the GTJ were found. It would be hoped that CARIES-QC could therefore identify changes which may occur as the result of different interventions for dental caries.

### Limitations

A key aim was to retain the language children used in the development stage in the definitive measure. Therefore, while the measure reflects the language used by children resident in Sheffield, it remains to be seen whether this language is appropriate outwith this geographical location. Attempts were made to ensure local colloquialisms were not included and certainly it would appear that the language the children used to describe pain was similar to that found throughout the UK and the US [65–67]. The CHU9D, a preference-based QoL measure, which was also developed in Sheffield, incorporates similar words in its response format and has been used successfully in other parts of the UK and Australia and New Zealand [68–70]. Nonetheless, it would be prudent to conduct preliminary evaluations before applying the measure in other English speaking countries.

CARIES-QC is designed for use with children in the age range of 5–16 years, as the impacts that children described were similar regardless of age. The items were worded using the language used by the youngest children to ensure that it could be understood by all. Further qualitative enquiry with children in the permanent dentition is required to assess whether items relating to aesthetic impacts would be a useful addition as this may be something which is more important to children in this older age group.

CARIES-QC had good construct validity and unidimensionality in this population. However, it is acknowledged that only two children were recruited from primary care, and the participants had a high caries prevalence and extensive disease. Although the majority of participants had extensive disease (80% had pulpal involvement), a number reported that they had not experienced pain. It is possible, therefore, that the findings may be different in a population with less extensive disease. However, it should be recognised that the measure was primarily designed to evaluate change in those who require interventions for dental caries and not to discriminate between children with different levels of disease. Therefore, it is likely that those who require treatment, will have some impacts which may improve following an intervention.

The ICC for test-retest reliability was slightly lower than ideal, and this may be due to the length of time between the two administrations of the measure, the low number of children who felt that their oral condition had remained stable or the fact that use of a three-point response scale may reduce test-retest reliability [64]. Testing of this element in a larger population with a shorter and more consistent time period between administrations is required.

Responsiveness was only tested in a very small proportion of the participants (*n* = 43) to give an indication of whether it responded as expected. However, the initial results are promising with those who felt they had improved having lower mean scores than those who did not report an improvement. An estimation of the MID was made for this population, however, additional testing in a larger population is required to further test the responsiveness of the measure. Larger populations would also allow the calculation of effect sizes and the standardised response mean, which would aid the clinical interpretation and calculation of future sample sizes [54].

## Conclusion

In conclusion, children’s input allowed the development of a valid, reliable and responsive child-centred caries-specific quality of life measure. CARIES-QC now offers a much needed tool to evaluate which interventions for dental caries are most effective in reducing impacts from the child’s perspective.

## Additional files

Additional file 1: Children’s suggestions for questionnaire design. (DOCX 137 kb)

Additional file 2: CARIES-QC fit to the Rasch model. (DOCX 56 kb)

Additional file 3: Correlations (Spearman’s rho) between CARIES-QC total scores and clinical data and CPQ_11–14_-ISF:16 total scores and clinical data. (DOCX 62 kb)

## Abbreviations

CARIES-QC: Caries Impacts and Experiences Questionnaire for Children
CCDH: Charles Clifford Dental Hospital
CI: Confidence interval
CIS: CARIES-QC interval scale
COHIP: Child Oral Health Impact Profile
C-OIDP: Child Oral Impacts on Daily Performances
COSMIN: Consensus-based Standards for the selection of health Measurement Instruments
CPQ: Child Perceptions Questionnaire
DMFT: Decayed, missing and filled permanent teeth
FG: Fiona Gilchrist, Principal Investigator
ICC: Intraclass correlation coefficient
IMD: Index of Multiple Deprivation
IRT: Item response theory
OHRQoL: Oral health-related quality of life
SD: Standard Deviation
