# Supplementary material for: Development and evaluation of CARIES-QC: a caries-specific measure of quality of life for children
Source: BMC Oral Health. 2018 Dec 4;18:202. doi: 10.1186/s12903-018-0662-8 (PMC6280387; doi:10.1186/s12903-018-0662-8)
Supplement: Supplementary file 1 — Children’s suggestions for questionnaire design. (DOCX 137 kb) [file 12903_2018_662_MOESM1_ESM.docx]

**Additional file 1**

**Stage 3: Children’s suggestions for questionnaire design.**

The first version children were shown (V1) contained a lead-in with three statements as response options. This type of response format has been used in the CHU9D and the CAT-QoL successfully [1, 2]. Participants selected their preferred option using a tick in a box. The second version children were shown (V2) had a statement with three response options which could be circled to indicate their answer. A similar response format is used in the Oxford Foot and Ankle Questionnaire for Children measure [3]. These can be seen in Figure 1. Children were asked to complete both questionnaires and state which format they preferred. They were also shown other designs and response formats. All but two of the children preferred V2 of the questionnaire, with one having no preference. All children felt that V2 was easier to read. The children also preferred V2 over the other examples they were shown. It was also suggested that “Please circle one answer” should be positioned at the top of each page to remind participants of what to do.

Finally children were asked if they thought coloured or white paper was best for the measure? The majority children preferred coloured paper as they felt it made the text stand out better (easier to read) and made the questionnaire look “more interesting”. They opted for the “gender-neutral” colour of purple.


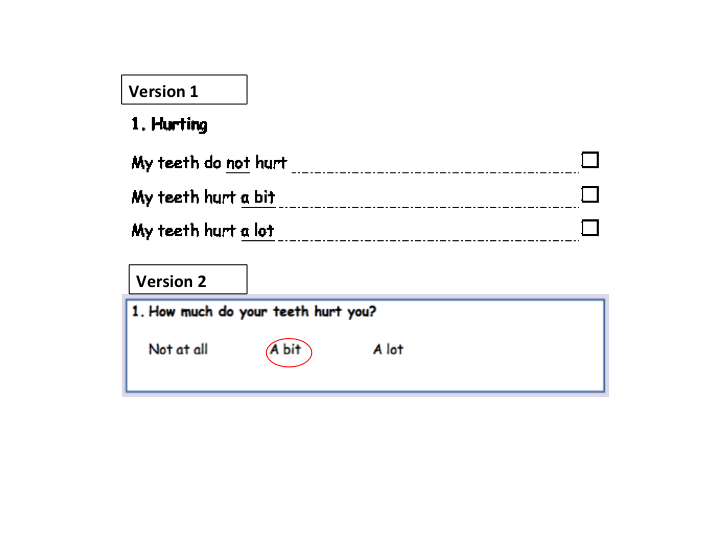


Figure 1: Questionnaire versions presented to children

1. Carlton J: **Developing the draft descriptive system for the child amblyopia treatment questionnaire (CAT-Qol): a mixed methods study**. *Health Qual Life Outcomes* 2013, **11**:174.

2. Stevens K: **Developing a descriptive system for a new preference-based measure of health-related quality of life for children**. *Quality of life research : an international journal of quality of life aspects of treatment, care and rehabilitation* 2009, **18**(8):1105-1113.

3. Morris C, Doll HA, Wainwright A, Theologis T, Fitzpatrick R: **The Oxford ankle foot questionnaire for children: scaling, reliability and validity**. *J Bone Joint Surg Br* 2008, **90**(11):1451-1456.
