# Supplementary material for: Development and evaluation of CARIES-QC: a caries-specific measure of quality of life for children
Source: BMC Oral Health. 2018 Dec 4;18:202. doi: 10.1186/s12903-018-0662-8 (PMC6280387; doi:10.1186/s12903-018-0662-8)
Supplement: Supplementary file 2 — CARIES-QC fit to the Rasch model. (DOCX 56 kb) [file 12903_2018_662_MOESM2_ESM.docx]

| Analysis name | Item residual | | Person residual | | Chi-square | | | Reliability | Unidimensionality | |
| --- | --- | --- | --- | --- | --- | --- | --- | --- | --- | --- |
|  | Mean | SD | Mean | SD | | Value (df) | P |  | Percentage of tests > 5% | 95% CI |
| Initial analysis | -0.39 | 1.62 | -0.23 | 1.04 | | 112 (32) | 0 | 0.86 | 4% | 1.96 – 14.04 |
| Remove item “tired” | -0.41 | 1.66 | -0.21 | 1.03 | | 92 (30) | 0 | 0.85 | 4.5% | 3 – 15.04 |
| Remove items “holes”, “brown” and “medicine” | -0.04 | 0.90 | -0.25 | 1.00 | | 39 (24) | 0.03 | 0.85 | 4% | 2 – 14.04 |
| Remove 7 participants with misfit | -0.37 | 0.86 | -0.21 | 0.89 | | 38 (24) | 0.04 | 0.85 | 4.15% | 2.07 – 13.93 |
| Ideal | 0 | 1 | 0 | 1 | |  | >0.004* | >0.7 | <5% | LCI <5 |

Additional file 2 CARIES-QC fit to the Rasch model.

*Bonferroni adjusted for 12 items; SD=standard deviation; df=degrees of freedom; CI=confidence interval; LCI=Lower confidence interval.
