# Supplementary material for: Development and evaluation of CARIES-QC: a caries-specific measure of quality of life for children
Source: BMC Oral Health. 2018 Dec 4;18:202. doi: 10.1186/s12903-018-0662-8 (PMC6280387; doi:10.1186/s12903-018-0662-8)
Supplement: Supplementary file 3 — Correlations (Spearman’s rho) between CARIES-QC total scores and clinical data and CPQ11–14-ISF:16 total scores and clinical data. (DOCX 62 kb) [file 12903_2018_662_MOESM3_ESM.docx]

| Clinical variable | CARIES-QC total score  (n = 197) | Transformed interval CARIES-QC total score  (n = 197) | CPQ_11-14_-ISF:16 total score  (n = 188) |
| --- | --- | --- | --- |
| Total number of carious teeth | 0.188** | 0.188** | 0.147* |
| Total dmft/DMFT | 0.129 | 0.129 | 0.121 |
| Pain | 0.392** | 0.392** | 0.312** |
| Pulpal involvement | 0.187** | 0.187** | 0.178* |
| Anterior caries | 0.154* | 0.147* | 0.048 |

Additional file 3 Correlations (Spearman’s rho) between CARIES-QC total scores and clinical data and CPQ_11-14_-ISF:16 total scores and clinical data.

|  |  |  |  |
| --- | --- | --- | --- |
| *p<0.05; **p<0.01; dmft = total number of decayed, missing and filled primary teeth; DMFT = total number of decayed, missing and filled permanent teeth. |  |  |  |
